# Leptin, adiponectin levels and their ratios in women with polyendocrine metabolic ovarian syndrome: a systematic review and meta-analysis

**DOI:** 10.3389/fmed.2026.1912483

**Published:** 2026-08-17

**Authors:** Xiaomeng Shi, Wenting Zhang

**Affiliations:** 1 Department of Gynecology, Chongqing Health Center for Women and Children, Chongqing, China; 2 Department of Gynecology, Women and Children's Hospital of Chongqing Medical University, Chongqing, China; 3 Chongqing Municipal Health Commission Key Laboratory of Perinatal Medicine, Chongqing, China; 4 NHC Key Laboratory of Birth Defects and Reproductive Health, Chongqing, China

**Keywords:** adiponectin, adiponectin-to-leptin ratio, leptin, meta-analysis, polycystic ovary syndrome, polyendocrine metabolic ovarian syndrome, systematic review

## Abstract

**Background:**

Polyendocrine metabolic ovarian syndrome (PMOS, formerly polycystic ovary syndrome [PCOS]) is a common endocrine and metabolic disorder. The global burden of PMOS shows an overall increasing trend. Recent studies have revealed significant alterations in serum leptin and adiponectin levels in patients with PMOS. However, the results are inconsistent.

**Objective:**

To compare the serum leptin and adiponectin levels, as well as the adiponectin-to-leptin (A/L) ratio, between women with PMOS (diagnosed according to the Rotterdam criteria) and healthy controls aged 18–49 years and to provide evidence for future screening strategies and the investigation of the pathogenesis of PMOS.

**Methods:**

A comprehensive search of “PubMed,” “Web of Science,” “Embase,” and “Cochrane Library” was conducted from January 1, 2015, to March 31, 2026. The authors independently reviewed all relevant clinical trials via systematic evaluation of abstracts and titles.

**Results:**

A total of 62 studies met the inclusion criteria. The pooling analysis of all relevant studies revealed that adult women with PMOS had higher serum leptin levels (standardized mean difference (SMD): 0.83; 95% confidence interval (CI): 0.59 to 1.07; *p* < 0.0001) and lower adiponectin levels (SMD: −1.06; 95% CI: −1.47 to −0.66; *p* < 0.0001) than women without PMOS. Heterogeneity across studies was considerable and not eliminated in subgroup analyses. The A/L ratio was statistically significantly lower in PMOS (SMD: −2.31; 95% CI: −4.02 to −0.55; *p* = 0.010).

**Conclusion:**

Women with PMOS aged 18–49 exhibit higher leptin levels, lower adiponectin levels and a lower A/L ratio. Further well-designed adequately powered prospective studies are needed to clarify the association between these markers and PMOS, including assessments of their sensitivity, specificity, and optimal cutoff values for predicting PMOS risk.

**Systematic review registration:**

The protocol was registered on https://www.crd.york.ac.uk/PROSPERO/view/CRD420261364408 (CRD420261364408).

## 1 Introduction

Polyendocrine metabolic ovarian syndrome (PMOS, formerly polycystic ovary syndrome [PCOS]) is a common endocrine and metabolic disorder affecting 10–13% of reproductive-aged women (1). The global burden of PMOS shows an overall increasing trend, with significant differences among different regions and countries (2). In 2020, approximately $43 billion was attributed to healthcare costs in the United States for PMOS-related obstetric complications and long-term diseases (3). Patients with PMOS are clinically characterized by oligo-anovulation, infertility, hyperandrogenism (such as hirsutism and acne), and metabolic dysfunctions (such as insulin resistance (IR) and obesity) (4). While the exact mechanisms of PMOS are not fully understood, adipose tissue dysfunction plays a key role in its pathophysiology by paving the way for metabolic dysregulation (5). Dysfunctional adipose tissue, characterized by altered adipokines secretion, has affected critical body functions including inflammation and insulin sensitivity along with energy metabolism (6).

Among adipokines, leptin and adiponectin have been most extensively studied. Leptin, a 167 amino acid peptide with a molecular weight of 16 kDa, was discovered in 1994 by cloning the obesity gene in mice (7, 8). It acts by binding and activating the leptin receptors in the central and peripheral nervous systems, thereby regulating appetite and food intake, bone mass, basal metabolism, proinflammatory immune responses, reproductive function and insulin secretion, among other processes (9). Adiponectin is a 30 kDa monomeric glycoprotein, which is initially reported in 1995, with insulin-sensitizing, anti-inflammatory, anti-apoptotic, anti-fibrotic, and proangiogenic properties (10, 11). Research on the serum leptin and adiponectin levels in PMOS patients remains marked by inconsistencies. Some studies have reported increased leptin and decreased adiponectin in PMOS patients (12–14), whereas others have shown different results (15–17). Variations in diagnostic criteria, small sample sizes, ethnic backgrounds, and measurement techniques probably account for this heterogeneity. Given the opposing effects of leptin and adiponectin on various biological processes, the adiponectin-to-leptin (A/L) ratio may be a more robust marker of metabolic dysfunction than individual adipokine measurements (13, 15).

Thus, we aimed to conduct this systematic review and meta-analysis to evaluate the serum leptin and adiponectin levels, as well as the A/L ratio, in women with PMOS (diagnosed according to the Rotterdam criteria) and in healthy controls, both aged 18–49.

## 2 Methods

### 2.1 Study design

This work was conducted according to the Preferred Reporting Items for Systematic Reviews and Meta-Analyses (PRISMA) guidelines (18). The study protocol was published in PROSPERO on April 20, 2026 (Registration number: PROSPERO 2026 CRD420261364408). The PRISMA checklist can be found in Supplemental Table S1.

### 2.2 Data sources and search strategy

A systematic search of published literature was conducted in the electronic databases of PubMed, Web of Science, Embase and Cochrane Library from January 1, 2015 through March 31, 2026, to identify studies reporting the association between PMOS and serum leptin and adiponectin levels, as well as the A/L ratio. The complete search strategy for all databases was provided in Supplemental Table S2. The search was limited to English language studies. The retrieved records were imported into EndNote version X9.2, and duplicate records were removed. Furthermore, we conducted a manual search of the reference lists of recent relevant systematic reviews and included trials to identify any potentially eligible trials not captured by our search. We assessed the full text of studies whose titles and abstracts could contain information regarding PMOS and serum leptin or adiponectin levels.

### 2.3 Inclusion and exclusion criteria

The inclusion criteria were as follows: (1) studies with cohort, cross-sectional, or case–control designs; (2) studies that used the Rotterdam 2003 criteria (19) to define PMOS; and (3) studies that either reported the mean and standard deviation (SD) or provided sufficient data to extract the mean and SD of total serum leptin or adiponectin levels in reproductive-age women with or without PMOS.

The exclusion criteria were as follows: (1) letters to the editor, case reports, conference abstracts, non-English studies, review articles, and intervention studies; (2) studies involving women aged <18 or >49 years, pregnant women, or nonhuman models; (3) duplicated population; (4) studies lacking a control group, and those that did not clearly specify the diagnostic criteria used for the diagnosis of PMOS; (5) studies involving fewer than 10 PMOS cases or controls; and (6) studies lacking sufficient information for data analysis or extraction.

### 2.4 Study selection and data extraction

Two investigators (Xiaomeng Shi and Wenting Zhang) screened the studies independently based on the predefined inclusion and exclusion criteria. Any disagreements were resolved through discussion. The following information was extracted from each study: the title, the first author, the publication year, the country where the study was conducted, the study design, the selection of controls, the matching criteria, the sample size, age, body mass index (BMI), and the values of leptin, adiponectin, and A/L ratio in PMOS patients and controls. In the case of any missing or unclear data, corresponding authors were contacted via email.

### 2.5 Quality assessment

The quality assessment was performed by using the Newcastle-Ottawa Scale (NOS) (20) for case–control studies and the adapted NOS for cross-sectional studies (21). A higher number of stars indicates higher study quality. Summary scores were assigned for low-to-moderate quality studies (≤6) and high quality studies (≥7). No studies were excluded based on the quality assessment.

### 2.6 Data synthesis and analysis

Statistical analyses were performed using R Studio, version 4.2.3. For continuous variables, the effect measure was expressed as the standardized mean difference (SMD) and its 95% confidence interval (CI) between the PMOS and control groups. We used the formulas proposed by Wan et al. to convert medians and interquartile ranges into means with SD (22). The SD was calculated for each group using the following formula where the standard error of the mean (SEM) was reported: SEM × √(sample size) (23). For studies that compared groups of women with different PMOS phenotypes to a control group, we combined the two PMOS subgroups into a single PMOS group using the formulas described in Section 6.5.2.10 of the Cochrane Handbook for Systematic Reviews of Interventions (version 6.5, updated August 2024) (24). A random effects model was used for the meta-analysis. Heterogeneity was evaluated using the chi-square test, and Cochran’s Q statistic (*p* < 0.01) and I^2^ statistic were reported. Low, moderate, and high heterogeneity were defined based on the results of the I^2^ test values at the cutoffs of 25, 50, and 75%, respectively (25). The sensitivity analysis was performed to estimate the influence of each study on the overall effect size in the meta-analysis by removing a different individual study each time. Publication bias was visually examined using a funnel plot, and Egger’s test was conducted to statistically assess the bias. Subgroup analyses were performed to detect the impact of BMI (BMI < 25 kg/m^2^ and BMI ≥ 25 kg/m^2^), study region (Africa, Asia, Europe, Turkey, and Oceania), study quality (low-to-moderate and high), study design (case–control study and cross-sectional study), year of publication (2015–2017, 2018–2020, 2021–2023, and 2024–2026), and sample size (<50, 50–100, 100–200, and >200) on study outcomes, only when each subgroup included at least two studies.

## 3 Results

### 3.1 Selection and characteristics of the included studies

**Figure 1:**
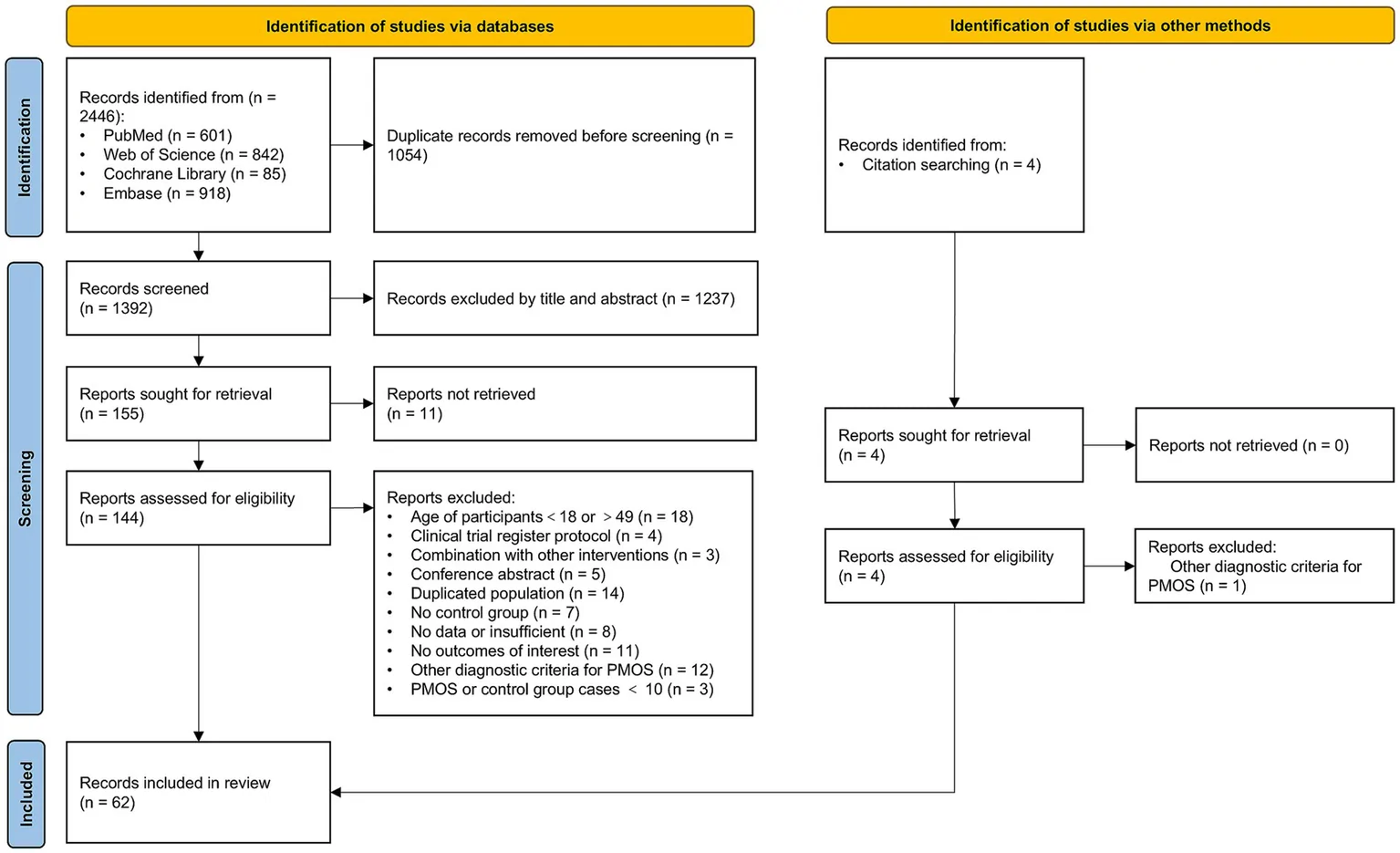
PRISMA chart showing the process of the selection of the included studies.

According to the described search strategy, a total of 2,446 studies were retrieved. Of those, 62 studies were identified to be eligible for this systematic review in the selection process (12, 15–17, 26–83). The screening process was shown in Figure 1.

The 62 studies consisted of 43 case–control studies and 19 cross-sectional studies, with no cohort studies. In the case–control studies, a total of 4,609 women with PMOS were compared with 3,673 women without PMOS. In the cross-sectional studies, a total of 1,281 women with PMOS were compared with 1,218 women without PMOS. The characteristics of the included case–control and cross-sectional studies are presented in Tables 1, 2, respectively.

**Table 1:** Characteristics of individual case-control studies included in the systematic review and meta-analysis.

Study	Country	Matching criteria	Outcome	Participants (PMOS/Controls)	Age (PMOS; controls)	BMI (PMOS; controls)
Dams (2021) (26)	Poland	Age matched	Leptin	77 (40/37)	29 [26–32]; 26 [24–29]	22.1 [20.7–26.1]; 20.9 [19.6–22.6]
Koleva (2016) (27)	Bulgaria	Age matched	Leptin and adiponectin	87 (67/20)	IR PMOS: 23.71 ± 5.88 Non-IR PMOS: 24.74 ± 4.81 Controls: 26.19 ± 5.1	IR PMOS: 28.53 ± 5.87 Non-IR PMOS: 25.88 ± 4.98 Controls: 26.14 ± 4.37
Gözüküçük (2020) (28)	Turkey	Age and BMI matched	Leptin and adiponectin	80 (40/40)	24.15 ± 3.570; 24.5 ± 3.289	23.18 ± 3.037; 23.3 ± 3.385
Vatannejad (2023) (12)	Iran	Age and BMI matched	Leptin and adiponectin	200 (150/50)	29.84 ± 3.99; 31.56 ± 3.68	25.79 ± 3.37; 25.35 ± 3.27
Li (2021) (33)	China	NR	Leptin	295 (114/181)	27.53 ± 4.14; 27.71 ± 4.17	23.82 ± 4.65; 21.22 ± 2.32
Obirikorang (2019) (34)	Ghana	NR	Leptin and adiponectin	156 (104/52)	Non-obese PMOS: 32.11 ± 4.25 Obese PMOS: 33.64 ± 4.14 Controls: 31.63 ± 4.88	Non-obese PMOS: 25.72 ± 2.95 Obese PMOS: 39.19 ± 2.99 Controls: 25.33 ± 2.68
Nambiar (2016) (35)	India	NR	Adiponectin	482 (282/200)	28.64 ± 5.06; 31.11 ± 5.13	25.12 ± 3.03; 23.49 ± 3.06
Patil (2022) (36)	India	Age and BMI matched	Adiponectin	90 (60/30)	23.69 ± 5.42; 24.80 ± 5.65	28.68 ± 7.52; 24.68 ± 6.00
Jahromi (2017) (38)	Iran	NR	Leptin	189 (99/90)	27.64 ± 4.34; 29.35 ± 5.95	26.47 ± 3.62; 24.82 ± 5.18
Kargasheh (2021) (39)	Iran	NR	Leptin	468 (324/144)	29.90 ± 4.56; 31.86 ± 5.12	26.70 ± 4.49; 25.42 ± 3.96
Mazloomi (2022) (40)	Iran	Age and BMI matched	Leptin	203 (104/99)	25.90 ± 4.13; 26.13 ± 5.01	26.95 ± 3.49; 26.50 ± 4.10
Kumawat (2021) (41)	India	Age matched	Leptin	180 (90/90)	24.45 ± 6.16; 23.18 ± 2.26	23.62 ± 3.92; 23.06 ± 1.02
Polak (2020) (42)	Poland	NR	Leptin	73 (39/34)	23 [21–27]; 26 [24–28]	23.54 [21.47–25.93]; 22.92 [20.64–24.9]
Jawad (2025) (46)	Iran	NR	Adiponectin	162 (108/54)	Infertile PMOS: 29.69 ± 4.37 RPL PMOS: 29.76 ± 4.50 Controls: 29.53 ± 4.42	Infertile PMOS: 25.90 ± 3.39 RPL PMOS: 26.11 ± 3.40 Controls: 25.32 ± 3.17
Talib (2025) (48)	Iraq	Age matched	Adiponectin	90 (45/45)	18–40	18.5–35
Sharma (2023) (49)	India	NR	Leptin and adiponectin	240 (120/120)	27.81 ± 2.37; 32.31 ± 4.61	28.03 ± 4.32; 24.32 ± 2.46
Suryapani (2024) (50)	India	NR	Leptin and adiponectin	170 (120/50)	26.14 ± 2.24; 20–40	23.16 ± 3.37; NR
Eroglu (2021) (51)	Turkey	Age and BMI matched	Adiponectin	138 (80/58)	Non-obese PMOS: 22.10 ± 4.41 Obese PMOS: 22.82 ± 5.26 Non-obese controls: 23.22 ± 3.32 Obese controls: 23.52 ± 4.25	Non-obese PMOS: 21.46 ± 2.14 Obese PMOS: 31.71 ± 4.51 Non-obese controls: 21.36 ± 1.92 Obese controls: 31.62 ± 5.55
Shivasekar (2024) (52)	India	Age matched	Adiponectin	253 (143/110)	23.91 ± 3.97; 25.7 ± 5.58	27.3 ± 3.72; 21.08 ± 1.83
Peng (2022) (53)	China	Age matched	Leptin	228 (89/139)	Normal-FSI PMOS: 31.50 [30.00–32.75] Hyper-FSI PMOS: 32.00 [30.00–34.00] Normal-FSI controls: 31.00 [29.00–33.00] Hyper-FSI controls: 31.00 [29.00–34.00]	Normal-FSI PMOS: 22.90 [21.53–24.30] Hyper-FSI PMOS: 25.60 [24.00–28.00] Normal-FSI controls: 12.27 [9.43–16.83] Hyper-FSI controls: 15.66 [14.02–19.52]
Basavaraj (2024) (55)	India	Age and BMI matched	Adiponectin	90 (60/30)	23.66 ± 1.34; 24.21 ± 3.25	26.54 ± 4.00; 22.56 ± 3.21
Khan (2025) (57)	Pakistan	Age matched	Leptin and adiponectin	180 (120/60)	26.4 ± 4.8; 27.1 ± 5.2	29.8 ± 5.1; 23.5 ± 3.3
Daghestani (2021) (58)	Saudi Arabia	Age and BMI matched	Leptin	200 (100/100)	25.04 ± 0.39; 24.67 ± 0.539	29.11 ± 0.608; 29.71 ± 0.759
Dixit (2025) (59)	India	NR	Leptin	100 (50/50)	27.92 ± 4.18; 24.28 ± 2.75	29.94 ± 3.65; 23.07 ± 1.41
Gupta (2017) (60)	India	NR	Leptin and adiponectin	439 (223/216)	20–35	26.46 ± 10.44; 26.02 ± 4.55
Tu (2017) (61)	China	NR	Leptin	609 (326/283)	28.03 ± 3.34; 28.95 ± 3.57	23.15 ± 4.13; 21.57 ± 3.13
Mohana (2021) (63)	Bangladesh	NR	Leptin and adiponectin	40 (20/20)	27.30 ± 1.29; 26.45 ± 0.91	29.47 ± 1.35; 23.71 ± 0.71
Shetty (2022) (64)	India	NR	Leptin	300 (150/150)	26.95 ± 5.34; 24.64 ± 5.89	25.43 ± 5.00; 21.91 ± 4.023
Zhou (2025) (65)	China	NR	Leptin	160 (80/80)	29.1 ± 5.7; 28.6 ± 6.3	23.1 ± 2.7; 22.6 ± 2.4
Shanaki (2018) (66)	Iran	NR	Adiponectin	171 (86/85)	29.93 ± 4.04; 29.79 ± 4.30	25.91 ± 3.58; 24.99 ± 3.20
Ożegowska (2020) (69)	Poland	Age and BMI matched	Adiponectin	162 (94/68)	27.0 [24.0–29.0]; 28.0 [26.0–30.0]	21.0 [20.0–22.6]; 20.5 [19.5–22.3]
Gargari (2015) (70)	Iran	Age and BMI matched	Leptin	60 (30/30)	25.83 ± 4.00; 26.06 ± 4.44	25.00 ± 3.61; 23.68 ± 3.07
Ozay (2016) (72)	Turkey	NR	Leptin	400 (250/150)	23.99 ± 4.63; 24.43 ± 4.39	24.32 ± 3.40; 23.44 ± 4.08
Erkan (2017) (73)	Turkey	Weight matched	Adiponectin	60 (44/16)	21.50 [20.00–24.00]; 22.50 [19.50–24.75]	22.50 [20.25–26.00]; 21.00 [20.00–23.00]
Siddiqui (2024) (74)	India	NR	Leptin	100 (50/50)	20.4 ± 2.7; 21.0 ± 2.5	23.1 ± 3.2; 22.6 ± 2.5
Rajendra (2023) (75)	India	Age matched	Adiponectin	80 (40/40)	37.53 ± 5.16; 34.82 ± 4.63	38.68 ± 5.72; 22.34 ± 2.03
Ali (2016) (76)	Iraq	BMI matched	Leptin	84 (50/34)	28.220 ± 0.786; 33.559 ± 0.937	30.504 ± 0.888; 31.081 ± 1.323
Bannigida (2020) (79)	India	NR	Adiponectin	200 (100/100)	18–40	Non-obese PMOS: 25.6 ± 2.53 Obese PMOS: 35.7 ± 3.05 Non-obese controls: 21.2 ± 4.86 Obese controls: 32.2 ± 4.39
Daghestani (2018) (80)	Saudi Arabia	Age and BMI matched	Leptin	251 (130/121)	25.61 ± 0.39; 24.67 ± 0.50	28.66 ± 0.58; 27.77 ± 0.73
Sarray (2015) (15)	Bahrain	Age matched	Leptin and adiponectin	426 (211/215)	28.6 ± 6.1; 27.5 ± 7.0	29.1 ± 6.7; 26.2 ± 5.3
Ali (2016) (81)	Iraq	Age and BMI matched	Leptin and adiponectin	89 (42/47)	28.83 ± 0.76; 33.11 ± 0.82	30.80 ± 0.87; 29.13 ± 0.80
Mayoofee (2024) (82)	Iraq	Age matched	Adiponectin	150 (100/50)	23.30 ± 4.66; 23.84 ± 4.80	27.22 ± 7.20; 22.84 ± 4.63
Montazerifar (2016) (83)	Iran	Age and BMI matched	Leptin and adiponectin	70 (35/35)	29.3 ± 5; 30.7 ± 4.3	28 ± 4.8; 26.5 ± 4.1

PMOS, polyendocrine metabolic ovarian syndrome; BMI, body mass index; NR, not reported; IR, insulin resistance; RPL, recurrent pregnancy loss; FSL, fasting serum insulin.

**Table 2:** Characteristics of individual cross-section studies included in the systematic review and meta-analysis.

Study	Country	Matching criteria	Outcome	Participants (PMOS/Controls)	Age (PMOS; controls)	BMI (PMOS; controls)
Boshku (2018) (29)	Macedonia	NR	Adiponectin	78 (61/17)	Normal weight PMOS: 24.17 ± 3.3 Obese PMOS: 24.32 ± 4.3 Controls: 26.52 ± 5.1	Normal weight PMOS: 21.9 ± 1.9 Obese PMOS: 31.63 ± 5.1 Controls: 20.95 ± 2.5
Momo (2022) (30)	Cameroon	Age and BMI matched	Adiponectin	64 (32/32)	26.7 ± 4.7; 27.1 ± 4.9	28.9 ± 1.4; 28.3 ± 1.2
Mishra (2022) (31)	India	Age and BMI matched	Leptin and adiponectin	120 (60/60)	27.5 ± 2.83; 27.83 ± 3.03	25.06 ± 2.42; 25.15 ± 2.32
Baldani (2019) (32)	Croatia	Age, BMI and WHR matched	Leptin and adiponectin	246 (151/95)	26.5 ± 6.0; 26.4 ± 2.7	24.8 ± 4.8; 23.3 ± 4.1
Hamid (2022) (37)	Pakistan	NR	Adiponectin	150 (110/40)	Overweight/fatty PMOS: 25 ± 6 Non-fatty PMOS: 26.5 ± 5 Overweight/fatty controls: 30 ± 6 Non-fatty controls: 27.5 ± 7.5	Overweight/fatty PMOS: 27.2 ± 3.2 Non-fatty PMOS: 21.5 ± 2.1 Overweight/fatty controls: 26 ± 1 Non-fatty controls: 19.4 ± 1.68
Daan (2016) (43)	The Netherlands	NR	Leptin and adiponectin	100 (68/32)	HA-PMOS: 28.5 [23.5–32.5] NA-PMOS: 28.8 [25.8–31.2] Controls: 34.5 [30.7–37.7]	HA-PMOS: 29.5 [23.3–35.6] NA-PMOS: 21.8 [19.8–22.2] Controls: 22.5 [21.2–24.5]
Glinianowicz (2015) (44)	Poland	NR	Leptin and adiponectin	154 (87/67)	25.4 ± 5.5; 25.7 ± 4.9	29.4 ± 8.8; 28.3 ± 7.0
Dawood (2018) (45)	Egypt	NR	Leptin	90 (45/45)	26.78 ± 3.45; 27.03 ± 3.21	21.81 ± 1.20; 22.07 ± 1.90
Çınar (2025) (17)	Turkey	BMI matched	Leptin	60 (30/30)	25.5 ± 4.53; 29.17 ± 8.91	28.08 ± 4.86; 27.09 ± 5.15
Ganie (2019) (47)	India	Age and BMI matched	Adiponectin	464 (144/320)	Vegetarian PMOS: 25.68 ± 3.81 Non-vegetarian PMOS: 26.13 ± 4.43 Vegetarian controls: 26.53 ± 5.99 Non-vegetarian controls: 26.57 ± 4.11	Vegetarian group: 24.94 ± 3.61 Non-vegetarian PMOS: 24.68 ± 3.45 Vegetarian controls: 23.96 ± 4.17 Non-vegetarian controls: 23.97 ± 3.63
Guney (2021) (54)	Turkey	Age matched	Adiponectin	86 (43/43)	31.0 ± 8.38; 28.42 ± 5.22	28.05 ± 5.77; 27.13 ± 4.44
Shorakae (2018) (56)	Australia	NR	Leptin	68 (46/22)	30 ± 6; 29 ± 8	29 ± 6; 33 ± 7
Jalilian (2016) (62)	Iran	NR	Leptin	76 (40/36)	27.15 ± 4.31; 29.3 ± 5.5	26.62 ± 4.03; 23.52 ± 2.53
Qu (2016) (67)	China	NR	Adiponectin	202 (102/100)	28 ± 6; 27 ± 4	24.37 ± 5.36; 20.53 ± 2.67
Silva (2026) (68)	Portugal	NR	Leptin	63 (20/43)	18–40	26.4 ± 6.2; 22.4 ± 4.9
Kohzadi (2020) (71)	Iran	NR	Adiponectin	80 (40/40)	30.70 ± 6.14; 32.02 ± 6.24	26.66 ± 4.24; 25.33 ± 3.15
Dhotre (2026) (77)	India	Age matched	Leptin	160 (80/80)	25.2 ± 4.1; 24.8 ± 3.7	27.1 ± 4.2; 23.4 ± 2.9
Beyazit (2021) (78)	Turkey	Age matched	Leptin and adiponectin	177 (91/86)	26.5 ± 6.2; 28.8 ± 7.2	26.0 ± 5.3; 24.1 ± 4.4
Nikolettos (2024) (16)	Greece	NR	Leptin and adiponectin	61 (31/30)	27.26 ± 5.11; 30.03 ± 6.36	27.35 ± 6.57; 22.71 ± 4.62

PMOS, polyendocrine metabolic ovarian syndrome; BMI, body mass index; NR, not reported; WHR, waist-to-hip ratio; HA, hyperandrogenic; NA, normoandrogenic.

In terms of study regions, 3 studies were conducted in Africa, 41 in Asia, 10 in Europe, 7 in Turkey, and 1 in Oceania. As for the outcomes, 24 studies only measured serum leptin levels, 20 only measured serum adiponectin levels, and 18 studies measured both serum leptin and adiponectin levels. In these 62 studies, 1 study were matched for age, BMI and waist-to-hip ratio (WHR), 15 for both age and BMI, 13 for age only, 2 for BMI only, and 1 for weight only. The mean age and BMI of women with PMOS ranged from 20.4 to 37.5 and from 21.2 to 38.68, respectively. The mean age and BMI of women without PMOS ranged from 21.0 to 34.8 kg/m^2^ and from 19.4 to 33.0 kg/m^2^, respectively.

### 3.2 Quality assessments

The evidence quality was assessed for each study and was summarized in Supplemental Table S3. Briefly, 42 studies were classified as low-to-moderate quality, and the remaining 20 studies were classified as high quality. The most common reason for the low-to-moderate quality of case–control studies was the lack of reporting of the non-response rate. Across cross-sectional studies, the most prevalent high risk of bias was that the study samples were not truly or even somewhat representative of the average in the target population.

### 3.3 Differences in leptin levels between PMOS and non-PMOS women

**Figure 2:**
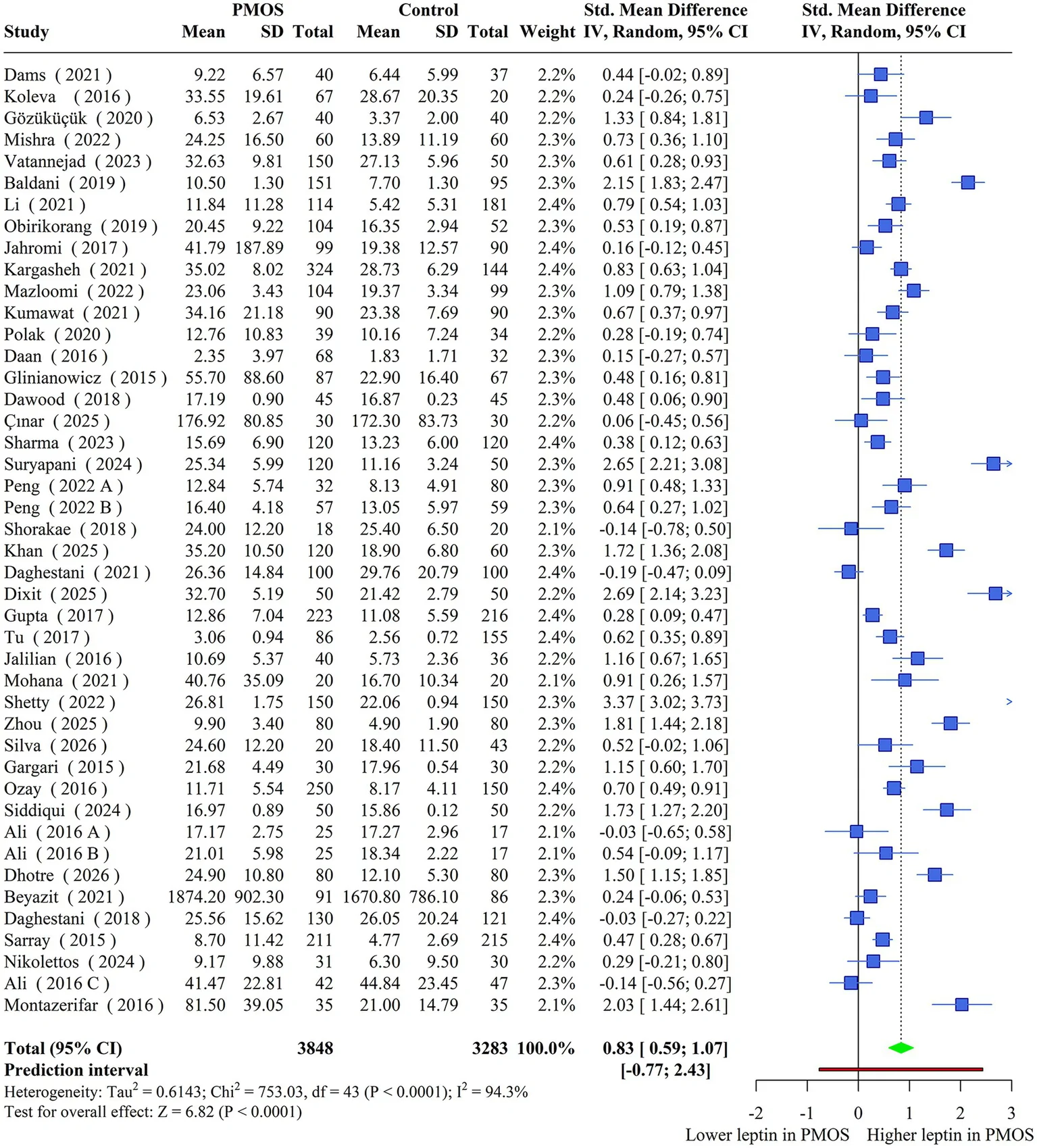
Forest plots of studies comparing leptin levels in women with and without PMOS.

A total of 42 studies (*n* = 7,131 women) assessed serum leptin levels in patients with and without PMOS. The forest plot for the relationship between PMOS and serum leptin levels was shown in Figure 2. The results indicated that women with PMOS had higher serum leptin levels than women without PMOS (SMD: 0.83; 95% CI: 0.59 to 1.07; *p* < 0.0001). However, high heterogeneity was observed among the studies (I^2^ = 94.3%; *p* < 0.0001). Subgroup analyses according to BMI, study region, study quality, study design, and year of publication revealed that significant differences maintained with high heterogeneity (Table 3). When studies were stratified by sample size, those with fewer than 50 participants demonstrated a non-significant reduction in leptin levels (SMD: 0.31; 95% CI: −0.17 to 0.80) with moderate heterogeneity (I^2^ = 56.5%). The leave-one-out sensitivity analysis, shown in Supplemental Figure S1, found that the pooled effect size remained stable regardless of the exclusion of any single study. The funnel plot in Supplemental Figure S2 was clearly asymmetric, suggesting significant publication bias. The Begg’s test and the Egger’s test did not observe statistically significant difference (z = 1.21, *p* = 0.225, and *t* = 1.58, *p* = 0.122, respectively). However, the presence of high heterogeneity reduced the power of these tests, and therefore the possibility of publication bias cannot be definitively excluded.

**Table 3:** Summary of SMD in leptin and adiponectin levels between PMOS and controls in different subgroup.

Subgroup	No. of reports	Random effect size SMD (95% Cl)	I^2^ (%)	*p* value for heterogeneity	*p* value for subgroup difference
Leptin					
BMI (kg/m^2^)					0.150
BMI < 25	9	0.75 [0.20; 1.30]	92.9	<0.0001	
BMI ≥ 25	8	0.40 [0.00; 0.80]	83.7	<0.0001	
Unknown	39	0.88 [0.61; 1.16]	94.6	<0.0001	
Region					0.133
Africa	2	0.51 [0.25; 0.77]	0.0	0.870	
Asia	27	0.99 [0.67; 1.31]	95.5	<0.0001	
Europe	8	0.58 [0.11; 1.05]	92.8	<0.0001	
Turkey	4	0.58 [0.05; 1.10]	84.7	0.000	
Study quality					0.480
High	14	0.73 [0.42; 1.03]	90.6	<0.0001	
Low-to-moderate	28	0.89 [0.56; 1.22]	95.3	<0.0001	
Study design					0.304
Case–control study	30	0.90 [0.61; 1.20]	95.0	<0.0001	
Cross-sectional study	12	0.65 [0.27; 1.03]	91.9	<0.0001	
Year of publication					0.068
2015–2017	13	0.54 [0.27; 0.81]	81.2	<0.0001	
2018–2020	7	0.67 [0.06; 1.27]	95.4	<0.0001	
2021–2023	13	0.81 [0.39; 1.24]	95.4	<0.0001	
2024–2026	9	1.45 [0.82; 2.07]	93.1	<0.0001	
Sample size					0.239
<50	3	0.31 [−0.17; 0.80]	56.5	0.075	
50–100	15	0.82 [0.41; 1.23]	90.2	<0.0001	
100–200	13	0.92 [0.59; 1.25]	94.3	<0.0001	
>200	11	0.96 [0.39; 1.53]	97.3	<0.0001	
Adiponectin					
BMI (kg/m^2^)					0.474
BMI < 25	10	−0.91 [−1.54; −0.28]	94.9	<0.0001	
BMI ≥ 25	9	−0.71 [−1.16; −0.26]	84.3	<0.0001	
Unknown	27	−1.17 [−1.75; −0.59]	97.1	<0.0001	
Region					0.004
Africa	2	−0.38 [−1.83; 1.08]	95.6	<0.0001	
Asia	24	−1.35 [−1.90; −0.80]	96.9	<0.0001	
Europe	7	−0.96 [−1.82; −0.11]	96.8	<0.0001	
Turkey	5	−0.12 [−0.52; 0.27]	76.3	<0.0001	
Study quality					0.577
High	11	−0.93 [−1.31; −0.55]	90.8	<0.0001	
Low-to-moderate	27	−1.13 [−1.73; −0.54]	97.3	<0.0001	
Study design					0.055
Case–control study	25	−1.31 [−1.88; −0.75]	97.0	<0.0001	
Cross-sectional study	13	−0.61 [−1.06; −0.15]	94.5	<0.0001	
Year of publication					0.024
2015–2017	10	−0.56 [−1.06; −0.07]	91.6	<0.0001	
2018–2020	9	−1.14 [−1.63; −0.66]	94.6	<0.0001	
2021–2023	11	−0.49 [−0.97; −0.01]	90.8	<0.0001	
2024–2026	8	−2.51 [−3.99; −1.03]	98.3	<0.0001	
Sample size					0.000
<50	2	0.20 [−0.27; 0.67]	0.0	0.391	
50–100	18	−0.74 [−1.12; −0.35]	90.1	<0.0001	
100–200	11	−1.73 [−2.80; −0.65]	97.5	<0.0001	
>200	8	−1.29 [−2.28; −0.30]	98.4	<0.0001	

SMD, standardized mean difference; PMOS, polyendocrine metabolic ovarian syndrome; CI, confidence interval; BMI, body mass index.

### 3.4 Differences in adiponectin levels between PMOS and non-PMOS women

**Figure 3:**
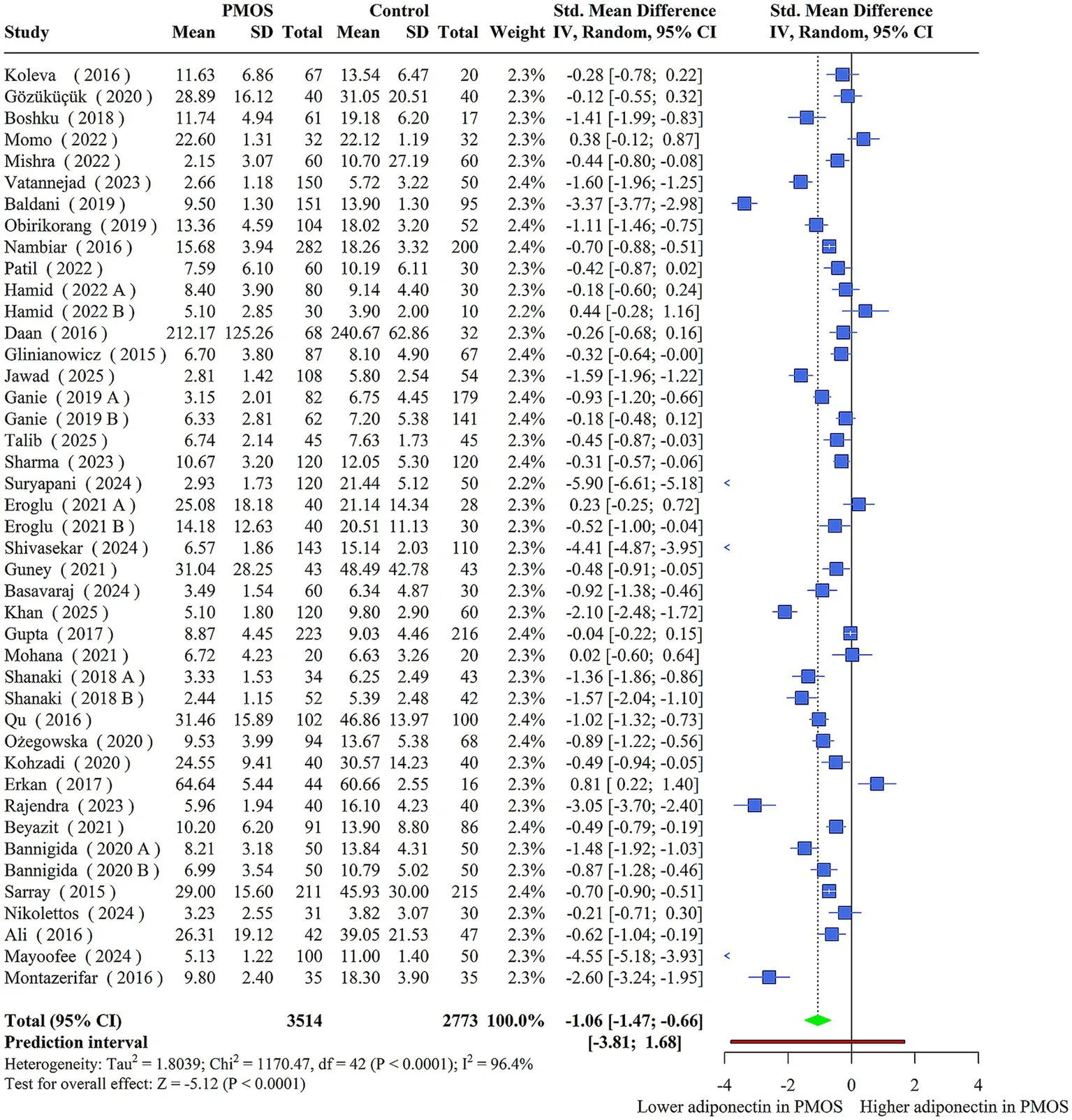
Forest plots of studies comparing adiponectin levels in women with and without PMOS.

A total of 38 studies (*n* = 6,287 women) assessed serum adiponectin levels in patients with and without PMOS. Figure 3 showed the forest plot for the correlation of PMOS with serum adiponectin levels. The results reported statistically significantly lower serum adiponectin levels among women with PMOS compared to those without PMOS (SMD: −1.06; 95% CI: −1.47 to −0.66; *p* < 0.0001). High heterogeneity was also observed among the studies (I^2^ = 96.4%). In the subgroup analyses according to BMI, study quality, study design, and year of publication, significant differences were maintained with high heterogeneity (Table 3). No significant difference was found when stratified by study region in the subgroup of studies conducted in Africa and Turkey (SMD: −0.38; 95% CI: −1.83 to 1.08 and SMD: −0.12; 95% CI: −0.52 to 0.27, respectively). No significant difference was found when stratified by sample size in the subgroup of studies with a sample size of less than 50 (SMD: 0.20; 95% CI: −0.27 to 0.67). The leave-one-out sensitivity analysis, shown in Supplemental Figure S3, revealed that the pooled effect size remained stable regardless of the exclusion of any single study. The funnel plot in Supplemental Figure S4 showed asymmetry, suggesting significant publication bias. This finding is consistent with the Egger’s test (*t* = −2.25, *p* = 0.030). However, the Begg’s test did not detect a statistically significant difference (z = −0.99, *p* = 0.320).

### 3.5 Differences in the A/L ratio between PMOS and non-PMOS women

**Figure 4:**
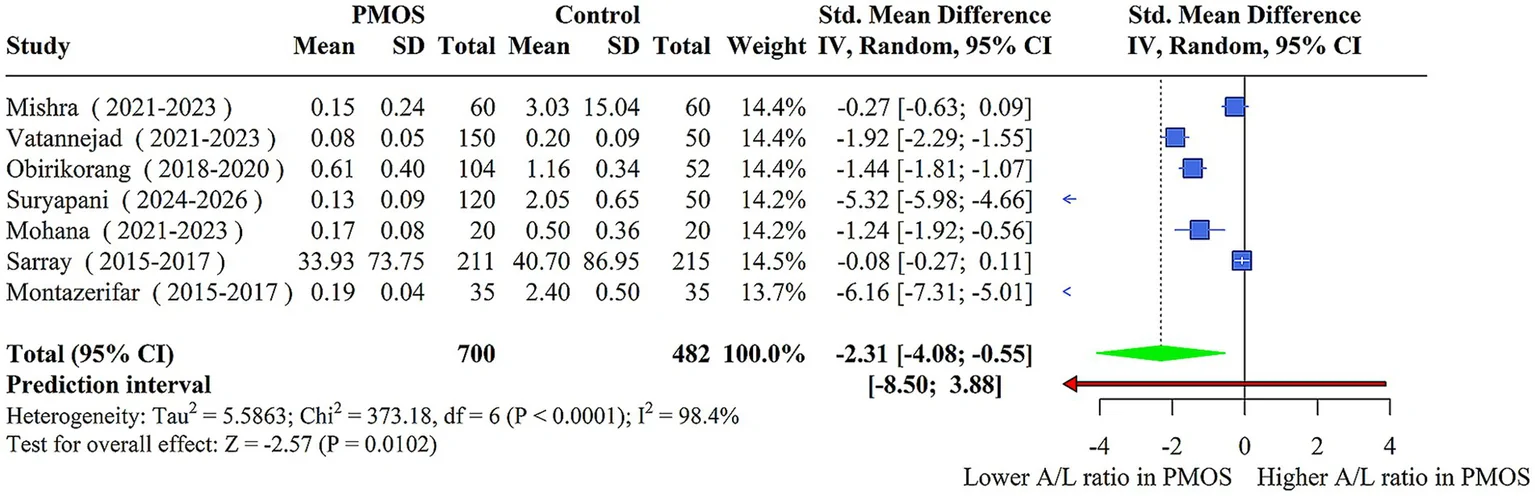
Forest plots of studies comparing the A/L ratio in women with and without PMOS.

A total of 7 studies (*n* = 1,182 women) assessed the A/L ratio in PMOS patients and controls. The forest plot for the link of PMOS with the A/L ratio was shown in Figure 4. There was a statistically significantly lower A/L ratio in PMOS (SMD: −2.31; 95% CI: −4.08 to −0.55; *p* = 0.010). The heterogeneity was also significant among the studies (I^2^ = 98.4%; *p* < 0.0001). The leave-one-out sensitivity analysis, shown in Supplemental Figure S5, revealed that the pooled effect size remained stable regardless of the exclusion of any single study. We did not perform subgroup analyses and did not assess the publication bias for the A/L ratio due to the limited number of included studies.

## 4 Discussion

Our main results revealed that, in women aged 18–49 with PMOS, there was a pro-inflammatory and IR profile of adipocytokines, characterized by elevated leptin, decreased adiponectin, and decreased A/L ratio. Our study indicated that these indicators may serve as robust biomarkers of PMOS.

PMOS is a complex endocrine and metabolic disorder, which has imposed a growing economic burden on society. The 2003 Rotterdam criteria define PMOS based on the presence of at least two of the following three features: oligo- or anovulation, clinical and/or biochemical signs of hyperandrogenism, and polycystic ovaries (19). Beyond its reproductive manifestations, PMOS is frequently accompanied by significant metabolic dysfunction, including insulin resistance, obesity, and adipocyte dysfunction (84). Adipose tissue exerts systemic metabolic effects through the secretion of multiple adipokines (85). Recent investigations have revealed significant alterations in the adipokine profile of patients with PMOS (14, 86, 87). Adipokine dysregulation may contribute to the pathogenesis of adipocyte dysfunction in PMOS (88).

Over the past decade, numerous studies have investigated serum leptin and adiponectin levels in patients with PMOS. However, the results are inconsistent. Some studies suggest that patients with PMOS have higher serum leptin levels and lower serum adiponectin levels than controls (12, 31, 89); however, some studies failed to find such an association (90, 91). To integrate these results, we conducted this meta-analysis to reach a reliable conclusion on the changes in serum leptin and adiponectin levels, as well as the A/L ratio, in PMOS patients aged 18–49. Given the heterogeneous nature of PMOS presentations, we limited our analysis to studies that used the Rotterdam 2003 criteria for diagnosis.

Leptin plays a role in the pathogenesis of PMOS through multiple pathways. It not only influences metabolism but also directly participates in reproductive function regulation. Obesity is notably prevalent among women with PMOS (92). It leads to higher leptin levels, which promote leptin resistance and thereby impair leptin’s regulatory effects on appetite, energy expenditure, and glucose homeostasis (93). At the neuroendocrine level, leptin acts on the hypothalamus to stimulate gonadotropin-releasing hormone (GnRH) neurons, modulating pulsatile activity to affect gonadotropin secretion and the LH surge (94). Leptin contributes to PMOS by inducing abnormal telomerase activity and telomere length in ovarian granulosa cells (65). A high dose of leptin affects FSH-induced steroidogenesis, suggesting that leptin may mediate negative rather than positive interactions with ovarian function, which may compromise fertility (95). In this study, we observed a statistically significant association between higher leptin levels and PMOS. Leptin dysregulation may play an important role in the development and progression of PMOS. Our findings are broadly consistent with previous meta-analyses that have similarly reported higher leptin levels in women with PMOS compared to controls (96, 97). Unlike previous meta-analyses, our study strictly restricted the age and PMOS diagnostic criteria of the included studies, which reduced confounding from phenotypic heterogeneity.

Adiponectin plays an important role in maintaining metabolic homeostasis (98). It functions as an insulin sensitizer and exhibits anti-diabetic, anti-inflammatory, and anti-atherogenic properties. Hyperandrogenism (HA) is recognized as a hallmark feature of PMOS. Elevated androgen levels can negatively impact adipocyte function and reduce adiponectin secretion (84). Lower adiponectin levels can exacerbate IR, leading to compensatory hyperinsulinemia (99). Hyperinsulinemia then enhances the release of kisspeptin, which amplifies androgen synthesis and worsens HA (100). This creates a pathological loop where both metabolic and reproductive issues persist (101). Adiponectin is a key negative regulator that links hyperandrogenism and IR. Our study found a statistically significant association between lower adiponectin levels and PMOS. Our results are consistent with those of the meta-analysis by Fernando et al. (102). They focused only on adolescent girls, whereas we focused on adult women aged 18–49 years. Our results suggest that hypoadiponectinemia is a long-standing feature of PMOS, providing evidence relevant to the diagnosis and pathological mechanism investigation of PMOS.

Changes in adipokine levels in patients with PMOS might be caused by obesity rather than the PMOS itself. Some studies have reported a positive correlation between leptin levels and obesity severity in PMOS (42, 87). Other studies have suggested an inverse correlation between adiponectin serum levels and BMI of PMOS (29, 103). To disentangle the independent effect of PMOS from that of obesity, we divided the participants into two groups based on the obesity status using a BMI cutoff of 25 kg/m^2^. PMOS was associated with increased leptin levels and decreased adiponectin levels in both the BMI < 25 kg/m^2^ and BMI ≥ 25 kg/m^2^ subgroups compared to the controls. These results indicated that dysregulated circulating leptin and adiponectin levels may play important roles in the occurrence and development of PMOS, independent of the degree of obesity.

In women with PMOS, leptin is positively correlated with insulin and Homeostatic Model Assessment of Insulin Resistance (HOMA-IR) (53, 104). Insulin and glucose measured during an oral glucose tolerance test (OGTT), along with a HOMA-IR score, are frequently used to evaluate IR, a key characteristic of PMOS, and can help to identify PMOS subtypes (105). Leptin has been shown to contribute to IR through the Janus kinase 2 and signal transducer and activator of transcription 3 (JAK2/STAT3) pathway (106). Deletion of leptin receptor in myeloid cells sufficiently improved IR and decreased systemic inflammation in diet-induced obese mice (107). Adiponectin also plays a role in IR. Some studies have shown that the serum adiponectin level is negatively related to IR in women with PMOS (108, 109). Adiponectin exerts anti-obesity, anti-diabetic, and insulin-sensitizing effects by stimulating lipid oxidation and enhancing insulin signaling (110). It exerts its metabolic effects through two specific receptors which activate key insulin-sensitizing signaling cascades, including the AMP-activated protein kinase (AMPK) and phosphoinositide 3-kinase/protein kinase B (PI3K/Akt) pathways (111). Reduced adiponectin levels, as observed in PMOS, may therefore facilitate the development of IR. Metformin can reverse the decrease in blood adiponectin associated with PMOS (112). Thus, accumulating evidence collectively suggests that leptin and adiponectin are promising targets in the treatment of PMOS.

Considering the inverse correlation between leptin and adiponectin in IR, the A/L ratio may be a more sensitive indicator of metabolic dysfunction than single adipokine measurements. Previous studies have found a negative correlation between the A/L ratio and serum amyloid A levels, which confirms that the A/L ratio may be used to estimate adipose tissue inflammation and, therefore, adipose tissue dysfunction (113). One study reported that A/L ratio was predictive of PMOS and was negatively associated with BMI and HOMA-IR, highlighting its potential prognostic value for cardiometabolic risk (15). Our study, integrating the results from seven studies, revealed that the A/L ratio was negatively correlated with PMOS. Further rigorous and well-designed studies are needed to investigate how adipokine dysregulation varies across PMOS phenotypes and its relationship with reproductive outcomes, thereby providing a more comprehensive understanding of the metabolic and endocrine features of the syndrome.

## 5 Strength and limitations

We conducted a comprehensive literature search strategy across multiple databases without restrictions by region or journal, allowing a broad assessment of the relationships between the serum leptin and adiponectin levels, as well as the A/L ratio, and PMOS. Due to the clinical heterogeneity of PMOS, we only included studies in which PMOS was diagnosed using the Rotterdam 2003 criteria. We limited the age range of the study population to 18–49 years to minimize the impact of age as much as possible. We performed sensitivity analysis and subgroup analyses to assess the potential impact of confounders.

Despite these strengths, this study has several limitations. First, substantial heterogeneity was observed across studies, raising concerns about the generalizability of the findings reported in this study. The high heterogeneity was not fully explained by subgroup analyses, suggesting the presence of unmeasured confounding factors such as the selection of controls, the methods used to measure adipokine levels, androgen levels, insulin resistance, and metabolic syndrome. Second, since the included studies were case–control or cross-sectional in design, our findings are limited to associations rather than causal inferences. Moreover, over half of the included studies were classified as low-to-moderate quality based on our systematic evaluation, which may have reduced the power of the meta-analyses. Additionally, all of the included studies were published only in English, which may contribute to potential language bias, as English studies are more likely to demonstrate positive findings.

## 6 Conclusion

In summary, this systematic review and meta-analysis showed higher serum leptin levels and lower serum adiponectin levels and A/L ratio in women aged 18–49 with PMOS diagnosed according to the Rotterdam criteria, although with high heterogeneity. These findings may help to elucidate disease mechanisms and candidate biomarker discovery. Further well-designed, adequately powered prospective studies are needed to clarify the association between these markers and PMOS, including assessments of their sensitivity, specificity, and optimal cutoff values for predicting PMOS risk.

## Data Availability

The datasets presented in this study can be found in online repositories. The names of the repository/repositories and accession number(s) can be found in the article/Supplementary material.
