# Supplementary material for: Leptin, adiponectin levels and their ratios in women with polyendocrine metabolic ovarian syndrome: a systematic review and meta-analysis
Source: Front Med (Lausanne). 2026 Aug 17;13:1912483. doi: 10.3389/fmed.2026.1912483 (PMC13526972; doi:10.3389/fmed.2026.1912483)
Supplement: Supplementary file 2 [file Table_2.DOCX]

Supplementary Table S2. Utilized search syntax, search fields, and filters across the electronic datasets.

**1. PubMed (601 records):**

| Search Keywords | Filters |
| --- | --- |
| ("Polycystic Ovary Syndrome" OR "Ovary Syndrome, Polycystic" OR "Syndrome, Polycystic Ovary" OR "Polycystic Ovarian Syndrome" OR "Ovarian Syndrome, Polycystic" OR "Polycystic Ovary Syndrome" OR "Sclerocystic Ovarian Degeneration" OR "Sclerocystic Ovary Syndrome" OR "Stein-Leventhal Syndrome" OR "Stein Leventhal Syndrome" OR "Syndrome, Stein-Leventhal" OR "Sclerocystic Ovaries" OR "Sclerocystic Ovary") AND ("leptin" OR "LEP" OR "adiponectin" OR "ADIPOQ" OR "APN" OR "ADI" OR "ADPN" OR "adipokin" OR "adipokines" OR "adipocytokines" OR "leptin adiponectin ratio" OR "leptin/adiponectin ratio" OR "L/A" OR "A/L" OR "adiponectin leptin ratio" OR "adiponectin/leptin ratio") | Search keywords were conducted through “All Fields”, applying the “English” language filter and limiting the publication date range from January 1, 2015 to March 31, 2026. |

**2. Web of Science (842 records):**

| Search Keywords | Filters |
| --- | --- |
| ("Polycystic Ovary Syndrome" OR "Ovary Syndrome, Polycystic" OR "Syndrome, Polycystic Ovary" OR "Polycystic Ovarian Syndrome" OR "Ovarian Syndrome, Polycystic" OR "Polycystic Ovary Syndrome" OR "Sclerocystic Ovarian Degeneration" OR "Ovarian Degeneration, Sclerocystic" OR "Sclerocystic Ovary Syndrome" OR "Stein-Leventhal Syndrome" OR "Stein Leventhal Syndrome" OR "Syndrome, Stein-Leventhal" OR "Sclerocystic Ovaries" OR "Ovary, Sclerocystic" OR "Sclerocystic Ovary") AND ("leptin" OR LEP OR "adiponectin" OR ADIPOQ OR APN OR ADI OR ADPN OR adipokin* OR adipokines* OR adipocytokines* OR "leptin adiponectin ratio" OR "leptin/adiponectin ratio" OR "L/A" OR "A/L" OR "adiponectin leptin ratio" OR "adiponectin/leptin ratio") | Search keywords were conducted through “All Fields”, applying the “English” language filter and limiting the publication date range from January 1, 2015 to March 31, 2026. |

**3. Cochrane Library (85 records):**

| Search Keywords | Filters |
| --- | --- |
| ("Polycystic Ovary Syndrome" OR "Ovary Syndrome, Polycystic" OR "Syndrome, Polycystic Ovary" OR "Polycystic Ovarian Syndrome" OR "Ovarian Syndrome, Polycystic" OR "Polycystic Ovary Syndrome" OR "Sclerocystic Ovarian Degeneration" OR "Ovarian Degeneration, Sclerocystic" OR "Sclerocystic Ovary Syndrome" OR "Stein-Leventhal Syndrome" OR "Stein Leventhal Syndrome" OR "Syndrome, Stein-Leventhal" OR "Sclerocystic Ovaries" OR "Ovary, Sclerocystic" OR "Sclerocystic Ovary") AND ("Visceral Adiposity Index" OR "Lipid Accumulation Product") | Search keywords were conducted through “Article title, Abstract, Keywords”, applying the “English” language filter and limiting the publication date range from January 1, 2015 to March 31, 2026. |

**4. Embase (918 records):**

| Search Keywords | Filters |
| --- | --- |
| ("Polycystic Ovary Syndrome" OR "Ovary Syndrome, Polycystic" OR "Syndrome, Polycystic Ovary" OR "Polycystic Ovarian Syndrome" OR "Ovarian Syndrome, Polycystic" OR "Polycystic Ovary Syndrome" OR "Sclerocystic Ovarian Degeneration" OR "Ovarian Degeneration, Sclerocystic" OR "Sclerocystic Ovary Syndrome" OR "Stein-Leventhal Syndrome" OR "Stein Leventhal Syndrome" OR "Syndrome, Stein-Leventhal" OR "Sclerocystic Ovaries" OR "Ovary, Sclerocystic" OR "Sclerocystic Ovary") AND ("leptin" OR LEP OR "adiponectin" OR ADIPOQ OR APN OR ADI OR ADPN OR adipokin OR adipokines OR adipocytokine OR adipocytokines OR "leptin adiponectin ratio" OR "leptin/adiponectin ratio" OR "adiponectin leptin ratio" OR "adiponectin/leptin ratio" OR "L/A" OR "A/L") | Search keywords were conducted through “Title, Abstract, Author Keywords”, applying the “English” language filter and limiting the publication date range from January 1, 2015 to March 31, 2026. |
