# Supplementary material for: Leptin, adiponectin levels and their ratios in women with polyendocrine metabolic ovarian syndrome: a systematic review and meta-analysis
Source: Front Med (Lausanne). 2026 Aug 17;13:1912483. doi: 10.3389/fmed.2026.1912483 (PMC13526972; doi:10.3389/fmed.2026.1912483)
Supplement: Supplementary file 3 [file Table_3.DOCX]

Supplemental Table S3. Quality assessment of included studies.

| **Newcastle - ottawa quality assessment scale for case-control studies** | | | | | | | | | | |
| --- | --- | --- | --- | --- | --- | --- | --- | --- | --- | --- |
| **Study** | **Section** | | | | **Comparability** | **Exposure** | | |  |  |
|  | **Is the case definition adequate?** | **Representativeness of the cases** | **Selection of Controls** | **Definition of Controls** | **Comparability of cases and controls on the basis of the design or analysis (Maximum: ☆☆)** | **Ascertainment of exposure** | **Same method of ascertainment for cases and controls** | **Non-Response rate** | **Score** | **Evidence quality** |
| Dams (2021) | ☆ | ☆ | ☆ | ☆ | ☆ | ☆ | ☆ |  | 7 | high quality |
| Koleva (2016) | ☆ | ☆ |  |  | ☆ | ☆ | ☆ |  | 5 | low-to-moderate quality |
| Gözüküçük (2020) | ☆ | ☆ |  |  | ☆☆ | ☆ | ☆ |  | 6 | low-to-moderate quality |
| Vatannejad (2023) | ☆ | ☆ |  | ☆ | ☆☆ | ☆ | ☆ |  | 7 | high quality |
| Li (2021) | ☆ | ☆ |  |  | ☆☆ |  | ☆ |  | 5 | low-to-moderate quality |
| Obirikorang (2019) | ☆ | ☆ | ☆ |  | ☆ | ☆ | ☆ |  | 6 | low-to-moderate quality |
| Nambiar (2016) | ☆ | ☆ |  | ☆ | ☆ | ☆ | ☆ |  | 6 | low-to-moderate quality |
| Patil (2022) | ☆ | ☆ |  |  | ☆ | ☆ | ☆ |  | 5 | low-to-moderate quality |
| Jahromi (2017) | ☆ | ☆ | ☆ |  | ☆ | ☆ | ☆ |  | 6 | low-to-moderate quality |
| Kargasheh (2021) | ☆ | ☆ |  | ☆ | ☆☆ | ☆ | ☆ |  | 7 | high quality |
| Mazloomi (2022) | ☆ | ☆ |  | ☆ | ☆☆ | ☆ | ☆ |  | 7 | high quality |
| Kumawat (2021) | ☆ | ☆ |  | ☆ | ☆ | ☆ | ☆ |  | 6 | low-to-moderate quality |
| Polak (2020) | ☆ | ☆ |  | ☆ | ☆ | ☆ | ☆ |  | 6 | low-to-moderate quality |
| Jawad (2025) | ☆ | ☆ |  | ☆ | ☆☆ | ☆ | ☆ |  | 7 | high quality |
| Talib (2025) | ☆ | ☆ |  | ☆ | ☆ | ☆ | ☆ |  | 6 | low-to-moderate quality |
| Sharma (2023) | ☆ | ☆ |  | ☆ | ☆ | ☆ | ☆ |  | 6 | low-to-moderate quality |
| Suryapani (2024) | ☆ | ☆ |  | ☆ |  | ☆ | ☆ |  | 5 | low-to-moderate quality |
| Eroglu (2021) | ☆ | ☆ |  | ☆ | ☆☆ | ☆ | ☆ |  | 7 | high quality |
| Shivasekar (2024) | ☆ | ☆ |  | ☆ |  | ☆ | ☆ |  | 5 | low-to-moderate quality |
| Peng (2022) | ☆ | ☆ |  | ☆ | ☆☆ | ☆ | ☆ |  | 7 | high quality |
| Basavaraj (2024) | ☆ | ☆ |  |  | ☆ | ☆ | ☆ |  | 5 | low-to-moderate quality |
| Khan (2025) | ☆ | ☆ |  | ☆ | ☆ | ☆ | ☆ |  | 6 | low-to-moderate quality |
| Daghestani (2021) | ☆ | ☆ |  | ☆ | ☆☆ | ☆ | ☆ |  | 7 | high quality |
| Dixit (2025) | ☆ | ☆ |  | ☆ |  | ☆ | ☆ |  | 5 | low-to-moderate quality |
| Gupta (2017) | ☆ | ☆ |  | ☆ |  | ☆ | ☆ |  | 5 | low-to-moderate quality |
| Tu (2017) | ☆ | ☆ |  | ☆ | ☆ | ☆ | ☆ |  | 6 | low-to-moderate quality |
| Mohana (2021) | ☆ | ☆ |  | ☆ | ☆ | ☆ | ☆ |  | 6 | low-to-moderate quality |
| Shetty (2022) | ☆ | ☆ |  | ☆ | ☆ | ☆ | ☆ |  | 6 | low-to-moderate quality |
| Zhou (2025) | ☆ | ☆ |  | ☆ | ☆☆ | ☆ | ☆ |  | 7 | high quality |
| Shanaki (2018) | ☆ | ☆ |  | ☆ | ☆☆ | ☆ | ☆ |  | 7 | high quality |
| Ożegowska (2020) | ☆ | ☆ |  | ☆ | ☆☆ | ☆ | ☆ |  | 7 | high quality |
| Gargari (2015) | ☆ | ☆ |  | ☆ | ☆☆ | ☆ | ☆ |  | 7 | high quality |
| Ozay (2016) | ☆ | ☆ |  | ☆ | ☆☆ | ☆ | ☆ |  | 7 | high quality |
| Erkan (2017) | ☆ | ☆ |  |  | ☆ | ☆ | ☆ |  | 5 | low-to-moderate quality |
| Siddiqui (2024) | ☆ | ☆ |  | ☆ | ☆☆ |  | ☆ |  | 6 | low-to-moderate quality |
| Rajendra (2023) |  | ☆ |  |  |  | ☆ | ☆ |  | 3 | low-to-moderate quality |
| Ali (2016 A) | ☆ | ☆ |  | ☆ | ☆ | ☆ | ☆ |  | 6 | low-to-moderate quality |
| Bannigida (2020) | ☆ | ☆ |  |  |  | ☆ | ☆ |  | 4 | low-to-moderate quality |
| Daghestani (2018) | ☆ | ☆ |  | ☆ | ☆☆ | ☆ | ☆ |  | 7 | high quality |
| Sarray (2015) | ☆ | ☆ | ☆ | ☆ | ☆☆ | ☆ | ☆ |  | 8 | high quality |
| Ali (2016 B) | ☆ | ☆ |  | ☆ | ☆ | ☆ | ☆ |  | 6 | low-to-moderate quality |
| Mayoofee (2024) | ☆ | ☆ |  | ☆ | ☆ | ☆ | ☆ |  | 6 | low-to-moderate quality |
| Montazerifar (2016) | ☆ | ☆ |  | ☆ | ☆☆ | ☆ | ☆ |  | 7 | high quality |

| **Adapted newcastle - ottawa quality assessment scale for cross-sectional studies** | | | | | | | | |
| --- | --- | --- | --- | --- | --- | --- | --- | --- |
| **Study** | **Study sample selection (Maximum: ☆☆)** | | **Assessment of exposure and outcome (Maximum: ☆☆☆☆)** | | **Confounding factors (Maximum: ☆☆☆)** | |  |  |
|  | **Representativeness of the study sample** | **Sample size** | **Ascertainment of the exposure(s)** | **Assessment of outcome(s)** | **Adjustment for confounder(s)** | **Assessment of confounder(s)** | **Score** | **Evidence quality** |
| Boshku (2018) | | ☆ | ☆☆ | ☆☆ |  |  | 5 | low-to-moderate quality |
| Momo (2022) | ☆ |  | ☆☆ | ☆☆ | ☆ | ☆ | 7 | high quality |
| Mishra (2022) | | ☆ | ☆☆ | ☆☆ | ☆ | ☆ | 7 | high quality |
| Baldani (2019) | |  | ☆☆ | ☆☆ | ☆ | ☆ | 6 | low-to-moderate quality |
| Hamid (2022) | |  | ☆☆ |  |  |  | 2 | low-to-moderate quality |
| Daan (2016) | |  | ☆☆ | ☆ | ☆ | ☆ | 5 | low-to-moderate quality |
| Glinianowicz (2015) | |  | ☆☆ | ☆☆ | ☆ | ☆ | 6 | low-to-moderate quality |
| Dawood (2018) | ☆ |  | ☆☆ |  |  |  | 3 | low-to-moderate quality |
| Çınar (2025) | |  | ☆☆ | ☆☆ | ☆ | ☆ | 6 | low-to-moderate quality |
| Ganie (2019) | | ☆ | ☆☆ | ☆☆ | ☆ | ☆ | 7 | high quality |
| Guney (2021) | |  | ☆☆ | ☆☆ | ☆ | ☆ | 6 | low-to-moderate quality |
| Shorakae (2018) | | ☆ | ☆☆ | ☆☆ | ☆ | ☆ | 7 | high quality |
| Jalilian (2016) | |  | ☆☆ | ☆☆ |  |  | 4 | low-to-moderate quality |
| Qu (2016) | |  | ☆☆ | ☆☆ |  |  | 4 | low-to-moderate quality |
| Silva (2026) | |  | ☆☆ | ☆☆ | ☆ | ☆ | 6 | low-to-moderate quality |
| Kohzadi (2020) | |  | ☆☆ | ☆☆ |  |  | 4 | low-to-moderate quality |
| Dhotre (2026) | |  | ☆☆ | ☆☆ |  |  | 4 | low-to-moderate quality |
| Beyazit (2021) | |  | ☆☆ | ☆☆ |  |  | 4 | low-to-moderate quality |
| Nikolettos (2024) | |  | ☆☆ | ☆☆ |  |  | 4 | low-to-moderate quality |
